# Investigations into the aetiopathogenesis of orofacial granulomatosis using multiple omics technologies reveal a potential role for B cells

**DOI:** 10.1002/ctm2.70689

**Published:** 2026-05-12

**Authors:** Maria Tumelty, Krupali Patel, Helen Petersen, Chris Delaney, David Lappin, John Gibson, Gordon Ramage, Christopher van der Gast, Andrew Smith, Christopher John Nile

**Affiliations:** ^1^ Oral Sciences Research Group, Uniersity of Glasgow Dental School University of Glasgow Glasgow UK; ^2^ Department of Microbial Diseases UCL Eastman Dental Institute University College London London UK; ^3^ Faculty of Biology Medicine and Health Division of Dentistry The University of Manchester Manchester UK; ^4^ Research Centre for Health Glasgow Caledonian University Glasgow UK; ^5^ Faculty of Science and Environment Northumbria University Ellison Place Newcastle upon Tyne UK; ^6^ School of Dental Sciences Framlington Place Newcastle upon Tyne UK; ^7^ Faculty of Medical Sciences Newcastle University; and NIHR Newcastle Biomedical Research Centre (BRC) Newcastle upon Tyne UK

**Keywords:** orofacial granulomatosis, B cells, rituximab, proximity extension assay, microbiome

1

Dear Editor,

Orofacial granulomatosis (OFG) is a granulomatous inflammatory disease of the oral cavity, with similarities to Crohn's disease (CD). OFG affects children and young adults, a proportion of whom have coexisting CD. Indeed, childhood‐onset OFG is a risk factor for CD. However, not all OFG patients develop CD.^1^

The aetiopathogenesis of OFG is unknown. Studies revealed that oral granulomatous diseases are genetically distinguishable from CD,^2^, ^3^ and microbial dysbiosis, including changes in abundance of *Streptococcus* and *Neisseria*, have been suggested to drive the granulomatous inflammatory response in OFG.^4^, ^5^ Alternatively, OFG has been described as an atopic condition and dietary intolerances implicated in its aetiology. However, not all patients see clinical improvements upon dietary exclusion.^5^ Studies into the immunopathogenesis of OFG are few and mainly histological. Tissue from patients with OFG has been found to contain a significantly higher number of CD3^+^ T cells, CD11c^+^ dendritic cells and B cells, including a novel subset of IgE‐expressing B cells, than tissue from patients with concurrent CD.^6^

Since OFG is an uncommon condition, there is a lack of robust clinical studies investigating its aetiopathogenesis. Therefore, 32 participants with OFG and 43 healthy controls were recruited, and multiple ‘omics’ technologies were applied to interrogate the aetiopathogenic mechanisms of disease (Figure 1A and Supporting Information Materials and Methods). There were no significant differences between demographic or clinical parameters between the study groups, including those with OFG alone and concurrent CD (Table S1).

**FIGURE 1:**
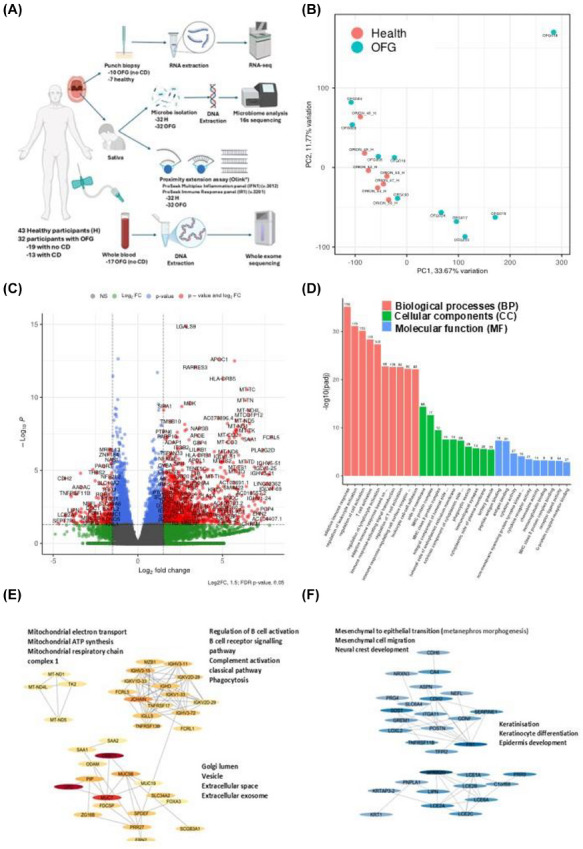
Overview of the study design and differential gene expression in biopsy tissue from participants with orofacial granulomatosis only compared to healthy controls. (A) Overview of study design (see Supporting Information). (B–F) Punch biopsy tissue samples from 10 participants with OFG alone (no concurrent CD), and seven healthy volunteers were analysed by RNA‐seq. The data have been deposited in NCBI's Gene Expression Omnibus and are accessible through GEO Series accession number GSE320069 (https://www.ncbi.nlm.nih.gov/geo/query/acc.cgi?acc = GSE320069). (B) Principal component analysis (PCA) revealed 33.67% of variance was attributed to the first component and 11.77% to the second component, and suggested that healthy participants clustered according to phenotype, while, in contrast, the OFG participants showed a heterogeneous spread in variation. (C) Volcano plot analysis of differentially expressed genes (DEGs). Scatter points represent genes. The x‐axis represents the log_2_ fold change in biopsy tissue from participants with OFG compared to healthy control tissue. The y‐axis represents the ‐log_10_ of the *p*_adj_‐value, in which 1.3 is equal to a *p*_adj_ of <0.05. There were 2304 differentially expressed genes (DEGs; 1714 upregulated and 590 downregulated) in the biopsy tissue of OFG patients, compared to biopsy tissue from healthy controls. (D) Gene ontology enrichment analysis (GO) to investigate the biological functions of the DEGs in biopsy tissues of participants with OFG revealed that enriched gene sets in OFG were predominantly associated with biological processes (BP) such as regulation of lymphocyte activation and T cell functions; cellular components (CC) such as MHC II protein complexes; and molecular functions (MF) such as antigen binding and cytokine receptor and chemokine activity. (E) STRING Protein‐Protein Interaction Networks Functional Enrichment Analysis of the top 50 upregulated genes (Table S1) attributed protein‐protein interaction pathways associated with B cell activity, phagocytosis and mitochondrial function. The nodes represent individual proteins, and the edges represent the protein‐protein interactions. (F) STRING Protein‐Protein Interaction Networks Functional Enrichment Analysis of the top 50 downregulated genes (Table S2) attributed protein‐protein interaction pathways associated with epithelial cell development from mesenchymal cells and keratinocyte differentiation and keratinisation. The nodes represent individual proteins, and the edges represent the protein‐protein interactions.

RNA‐seq analysis revealed that participants with OFG alone showed a heterogeneous spread in variation (Figure 1B) with 2304 differentially expressed genes (DEGs) (Figure 1C). Gene ontology enrichment analysis revealed the DEGs to be predominantly associated with adaptive immune responses (Figure 1D). STRING analysis of the top 50 up‐ (Table S2) and down‐ (Table S3) regulated genes identified upregulation of pathways associated with B cell activity and phagocytosis (Figure 1E) and downregulation of pathways associated with keratinocyte function (Figure 1F).

Whole Exome Sequencing revealed the most frequent mutations associated with OFG, ranked on allele frequency (AF), were found in the *KIAA2018* and *TAS2R31* genes. However, based on the Combined Annotation‐Dependent Depletion (CADD) score, mutations in the *SRGAP2* and *AK2* genes had a greater potential impact on disease (Table S4).

Analysis of the salivary immunoproteome revealed that 41.79% of variance was attributed to the first component and 13.08% to the second component (Figure 2A), and 55 proteins were significantly elevated in the saliva of patients with OFG (Figure 2B and Table S5). However, no significant differences in levels were determined between participants with OFG and those with concurrent CD (data not shown). STRING analysis identified upregulated pathways associated with regulation of adaptive immune responses, TNF and cytokine production (Figure 2C,D). ELISA analysis confirmed IL‐6 (Figure 2E), CXCL9 (Figure 2F) and CCL3 (Figure 2G) were elevated in all participants with OFG compared to healthy control participants (all *p* < 0.01). However, there was no significant difference in levels between participants with OFG and those with concurrent CD. There were also significant correlations (all *ρ* > 0.7) between ELISA levels and Olink NPX values (Figure 2H).

**FIGURE 2:**
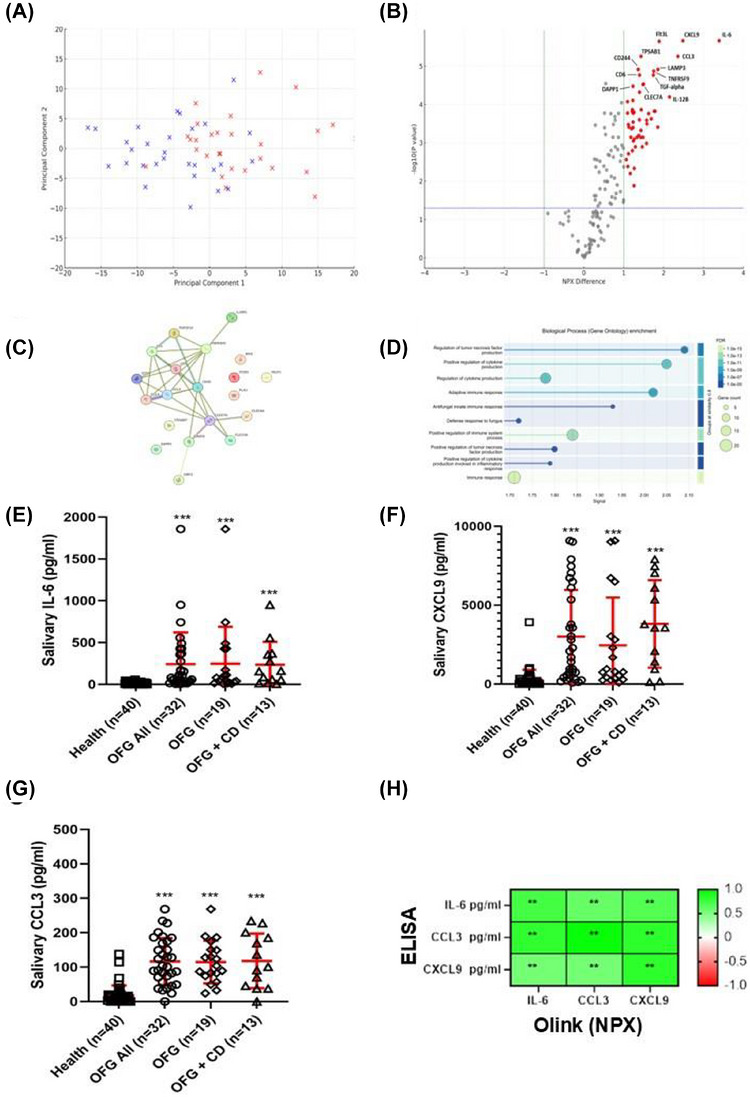
Differential levels of salivary immunoregulatory proteins between participants with orofacial granulomatosis and healthy controls. The salivary immunoregulatory protein profile was determined using proximity extension assay (PEA) analysis by Olink Proteomics (Uppsala, Sweden) (see Supporting Information). Analysis was performed using two panels: the ProSeek Multiplex Inflammation panel (IFN1) (v.3012) and the ProSeek Immune Response panel (IR1) (v.3201). In combination, this allowed for interrogation of salivary levels of 184 different proteins associated with the immune response (four proteins were present on both panels: CCL11, IL‐5, IL‐6 and IL‐10). The data is derived from an analysis of 29 saliva samples from the OFG all cohort and 29 healthy control saliva samples. (A) Principal component analysis revealed 41.79% of variance was attributed to the first component and 13.08% to the second component. (B) Volcano plot showing differential salivary levels of immunoregulatory proteins in OFG participants compared to healthy controls. Scatter points represent individual proteins, and select key upregulated proteins are labelled. The x‐axis represents the relative quantification (NPX) difference between the saliva of participants with OFG and healthy controls. An NPX value of 1 was used as a cut‐off to determine upregulation. The y‐axis represents the ‐log_10_ of the *p*_adj_‐value; 1.3 is equal to a *p*_adj_ of <0.05, which was used as a cut‐off to determine significant upregulation. In total, 55 immunoregulatory proteins were significantly upregulated in the saliva of participants with OFG compared to healthy controls. No immunoregulatory proteins were significantly downregulated. (C) STRING protein‐protein interaction network analysis was performed to determine the functional relationships between the top 20 upregulated salivary proteins in OFG participants (Table S5). The nodes represent individual proteins, and the edges represent the protein‐protein interactions. (D) Functional enrichment visualisation showing the top biological processes associated with the protein‐protein interactions in the saliva of participants with OFG. The elevated salivary proteins are attributed to protein‐protein interaction pathways associated with the regulation of TNF production, regulation of cytokine production and adaptive immune responses. (E) Salivary levels of IL‐6 for all study participants determined by conventional ELISA analysis. (F) Salivary levels of CCL9 for all study participants determined by conventional ELISA analysis. (G) Salivary levels of CCL3 for all study participants determined by conventional ELISA analysis. For E – G, *n* is shown on graphs. *** p<0.001 compared to the healthy control group. (H) Spearman's rho correlation analysis between the absolute quantification of salivary levels of IL‐6, CCL3 and CXCL9, as determined by ELISA, and the NPX determined by Olink. **p < 0.01.

Faecal calprotectin (MRP8/14; S100A8/9) is used in both the diagnostic workup and monitoring of patients with IBD. In a paediatric population with early onset IBD, salivary calprotectin levels were significantly elevated in those with OFG, compared to those with IBD alone.^7^ Analysis of salivary calprotectin revealed levels that were significantly elevated in all participants with OFG (*p* < 0.001), but there was no difference between participants with OFG and those with concurrent CD (Figure 3A). In addition, salivary calprotectin levels strongly correlated with levels of IL‐6 and CXCL9 but no other parameters (Figure 3B).

**FIGURE 3:**
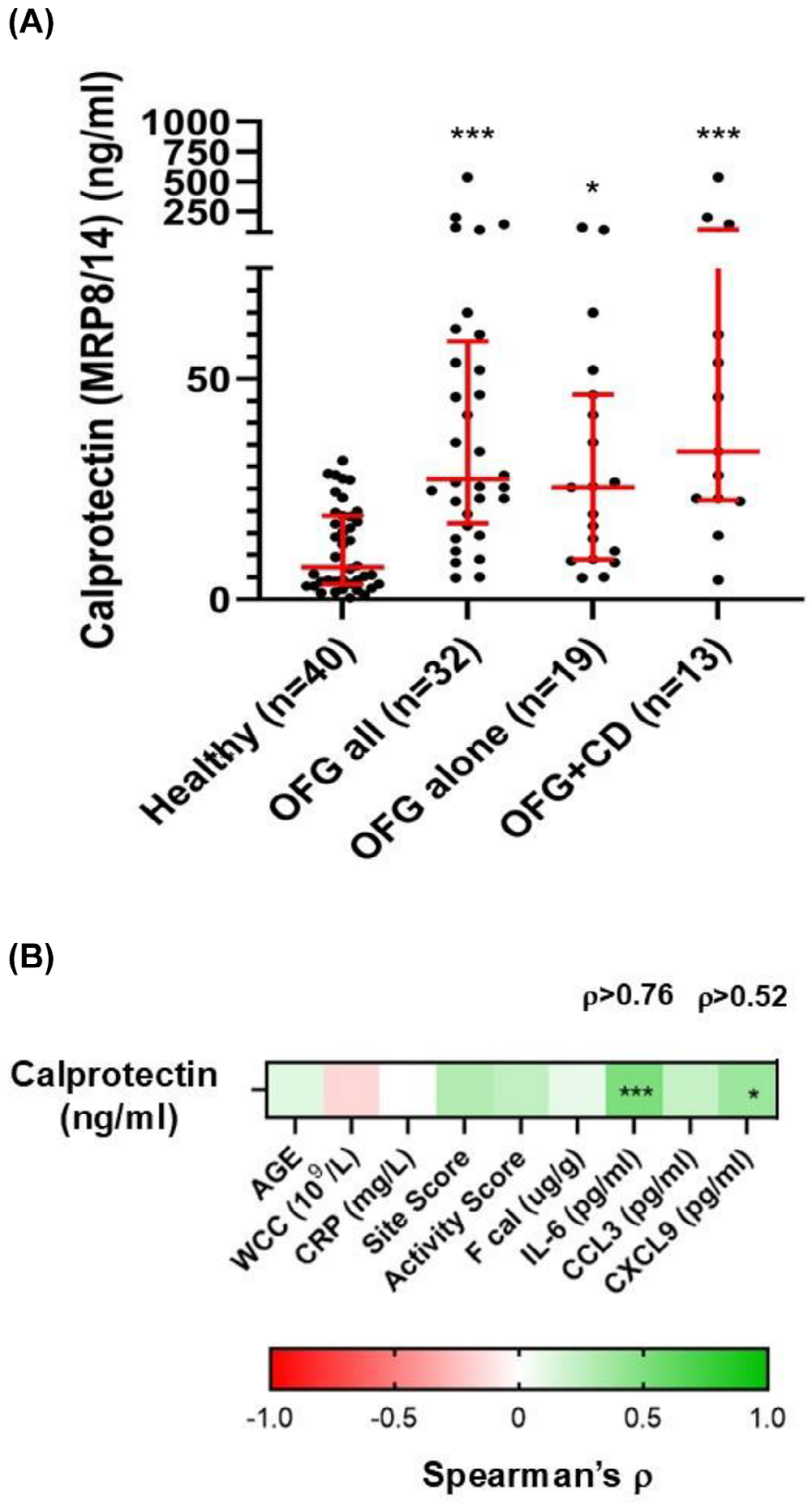
Differential levels of Salivary calprotectin (MRP8/14; S100A8/9) between participants with orofacial granulomatosis compared to healthy controls. (A) Salivary levels of calprotectin of all study participants determined using the calprotectin (MRP8/14; S100A8/9) assay kit (Buhlmann Laboratories AG, Switzerland) (see Supporting Information). *n* is shown on the graph. * *p* < 0.05, *** *p* < 0.001, compared to the healthy control group. Salivary levels of calprotectin were significantly elevated in all participants with OFG (*p* < 0.001), those with OFG alone (*p* ≤ 0.05) and those with OFG and concurrent CD (*p* < 0.001), compared to healthy control participants (Figure 4A). However, there was no significant difference in salivary levels between participants with OFG alone and those with concurrent CD (OFG+CD). (B) Spearman's rho correlation analysis between salivary levels of calprotectin and age of participants, selected haematological markers of inflammation, disease activity scores, faecal calprotectin (Fcal) and salivary levels of IL‐6, CCL3 and CXCL9. * *p* < 0.05, *** *p* < 0.001. Spearman's rho correlation analysis showed that salivary calprotectin levels exhibited a significant (*p* < 0.001) very strong correlation with salivary levels of IL‐6 (*ρ* > 0.76) and a significant (*p* < 0.05) strong correlation with salivary levels of CXCL9 (*ρ* > 0.52). However, no statistically significant correlations were determined between salivary levels of calprotectin and any other parameters.

Analysis of the salivary microbiome was performed to determine if dysbiosis contributed to the aetiopathogenesis of OFG. Using ecological principles (see Supporting Information), the OFG cohort had 185 core taxa and 724 satellite taxa. The core taxa accounted for 98.0% abundance. The healthy cohort had 216 core and 703 satellite taxa. The core taxa accounted for 99.6% abundance (Figure 4A). Diversity was significantly higher in the healthy cohort, compared to the OFG cohort, for the whole microbiota, core and satellite taxa (*p* < 0.05 in all instances) (Figure 4B). The compositional similarity between the OFG and healthy control cohorts was significantly different for the whole microbiota, core and satellite taxa (*p* < 0.0001 in all instances) (Figure 4C). SIMPER analysis suggested that *Streptococcus* and *Neisseria* were contributing most to the dissimilarity between cohorts (Table S6).

**FIGURE 4:**
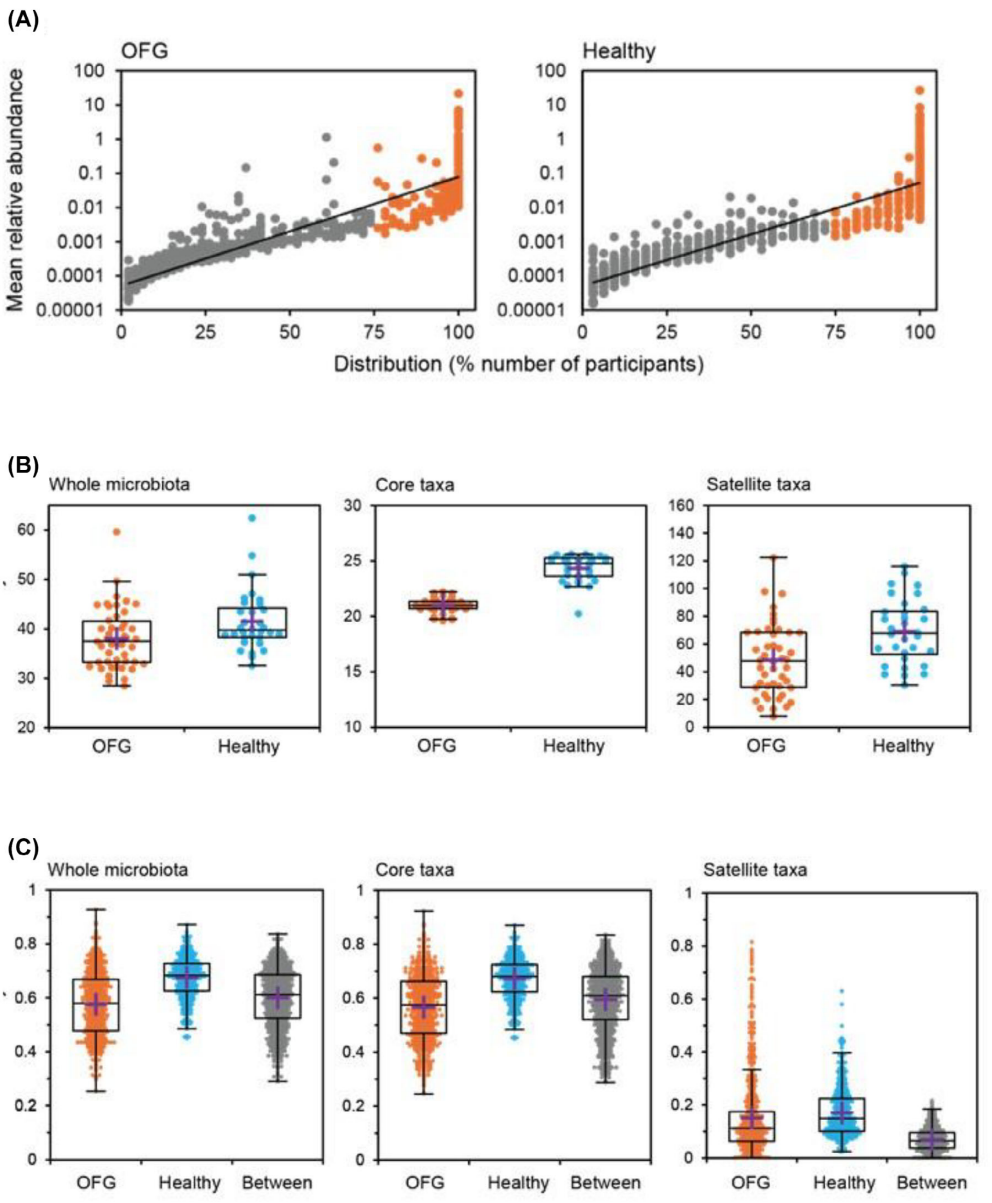
Comparison of the salivary microbiome between participants with orofacial granulomatosis and healthy controls. Analysis of the salivary microbiome was performed by Novogene (Novogene, UK) on the Illumina MiSeq platform (250 nucleotides, paired‐end) on 32 participants with OFG and 32 healthy controls (see Supporting Information). The sequencing data is available at PRJNA1429289 Temporary Submission ID: SUB16015641. (A) Distribution and abundances of bacterial taxa across OFG and healthy control participants. Given is the percentage number of participant samples each bacterial taxon was observed to be distributed across, plotted against the mean percentage abundance across those samples. Core taxa are defined as those that are within the upper quartile of distribution (≥75% of samples) (orange circles), and satellite taxa (grey circles) are defined as those that do not. Distribution‐abundance relationship regression statistics: OFG *R* ^2^ = 0.86, *F*_1907_ = 5410.4, *p* < 0.0001 and Healthy *R* ^2^ = 0.85, *F*_1917_ = 5336.9, *p* < 0.0001. (B) Comparison of diversity in the whole microbiota and the core and satellite taxa groups from within the OFG and healthy control participants. Diversity was measured using Fisher's alpha index of diversity. Boxplots show 25–75th interquartile (IQR) range with whiskers showing 1.5 times IQR. Purple crosses represent the mean in each group. Kruskal‐Wallis tests: Whole microbiota *H* = 7.247, *p* = 0.007; Core taxa *H* = 49.992, *p* < 0.0001; and Satellite taxa *H* = 11.546, *p* = 0.001. *H* critical was 3.841. (C) Comparison of similarity within and between OFG and healthy participants for the whole microbiota and the core and satellite taxa groups. Compositional similarities were measured using the Bray‐Curtis index of similarity. Boxplots show 25–75th IQR range with whiskers showing 1.5 times IQR. Purple crosses represent the mean in each group. Circles are pairwise similarities between compared samples within each instance. Kruskal‐Wallis tests assessing levels of similarity: Whole microbiota *H* = 267.293, *p* < 0.0001; Core taxa *H* = 253.171, *p* < 0.0001; and Satellite *H* = 69.516, *p* < 0.0001. *H* critical = 3.841. One‐way PERMANOVA tests (with Bonferroni correction) of compositional similarity between participant cohorts: Whole microbiota *F* = 5.105, *p* < 0.0001; Core taxa *F* = 5.242, *p* < 0.0001; and Satellite taxa *F* = 0.7582, *p* < 0.0001.

In terms of clinical presentation, salivary biomarkers and the oral microbiome, there were no differences between participants with OFG alone and those with concurrent CD. Microbiome analysis, in agreement with previous studies, implicated *Streptococcus* and *Neisseria* species in the aetiopathogenesis of OFG.^3^, ^4^ Interestingly, *Neisseria subflava*, from patients with OFG, was demonstrated to elicit granulomatous inflammation.^4^ Therefore, dysbiosis may play a role in driving granulomatous inflammation in patients with OFG. However, to position organisms as specific aetiological agents requires further studies.

The data show that B‐cell responses may play a role in OFG immunopathogenesis. RNAseq revealed a significant role for B cell genes and signalling pathways. In addition, WES identified mutations in *SRGAP2* and *AK2* that may predispose to OFG. Interestingly, *SRGAP2* is expressed in immune cells (https://v23.proteinatlas.org), but its function remains unknown. However, AK2 plays an important role in B cell immunometabolism, activation and antibody production.^8^ Furthermore, IL6 (B cell stimulatory factor 2), CXCL9 and CCL3 were elevated in the saliva of participants with OFG, all of which play a role in B cell maturation, viability, migration, differentiation into plasma cells and B cell receptor signalling.^9^ Adding to the evidence that B cells may play a role in OFG, a recent case study reported the use of the B‐cell‐depleting monoclonal antibody Rituximab as a treatment, which revealed promising results.^10^ Therefore, large‐scale, well‐controlled clinical studies using Rituximab may reveal more about the role of B cells in OFG immunopathogenesis and determine if targeting B cells is a treatment option for OFG.

## AUTHOR CONTRIBUTIONS

Christopher John Nile, John Gibson and Andrew Smith conceived and supervised the study. Maria Tumelty, Christopher John Nile, John Gibson, Gordon Ramage and Andrew Smith designed the research methodology. Maria Tumelty, David Lappin, Krupali Patel and Helen Petersen collected the data and performed the experiments. Maria Tumelty, Christopher John Nile, David Lappin, Chris Delaney and Christopher van der Gast conducted the formal analysis. Christopher John Nile, Maria Tumelty, Christopher van der Gast and Andrew Smith wrote the original draft of the manuscript. All authors contributed to data interpretation, critically revised the manuscript and approved the final version.

## CONFLICT OF INTEREST STATEMENT

The authors declare no conflict of interest.

## DECLARATION OF GENERATIVE AI AND AI‐ASSISTED TECHNOLOGIES IN THE MANUSCRIPT PREPARATION PROCESS

The authors declare that AI technologies were not used in the preparation of this work.

## ETHICS STATEMENT

Thirty‐two participants with OFG, with and without CD, attending Glasgow Dental Hospital & School's Department of Oral Medicine were recruited into the study.

## Supporting information



Supporting Information

Supporting Information

Supporting Information

Supporting Information

Supporting Information

Supporting Information

Supporting Information

## Data Availability

Data sharing not applicable to this article as no datasets were generated or analyzed during the current study.
