# Supplementary material for: Investigations into the aetiopathogenesis of orofacial granulomatosis using multiple omics technologies reveal a potential role for B cells
Source: Clin Transl Med. 2026 May 12;16(5):e70689. doi: 10.1002/ctm2.70689 (PMC13162125; doi:10.1002/ctm2.70689)
Supplement: Supplementary file 1 — Supporting Information [file CTM2-16-e70689-s006.docx]

**Supplementary Materials and Methods**

***Study cohorts***

Thirty-two participants with OFG, with and without CD, attending Glasgow Dental Hospital & School’s Department of Oral Medicine were recruited into the study. The study protocol was approved by the West of Scotland Research Ethics Service (16/WS/0159). Inclusion criteria included: new patients with suspected OFG and patients with diagnosed OFG, with and without CD. Exclusion criteria included: Any other known oral diseases (with particular reference to apical periodontitis, periodontal disease and caries), pregnancy, breast feeding, previous radiotherapy for the treatment of head and neck malignancy, patients unable to give consent due to incapacity and patients less than 5 yrs of age. Full details of the patient’s medical status were recorded which included reporting of any underlying CD and oral clinical status was determined using the Oral Disease Severity Scoring System (1). Exclusion criteria included: pregnancy, breast feeding, previous radiotherapy for the treatment of head and neck malignancy, steroid or immunosuppressive therapy in last 6 months, antibiotic therapy in the last 6 weeks and ongoing periodontal or restorative treatment.

Forty-three healthy control volunteers were recruited from the pool of students and staff at Glasgow University Dental School. The study protocol was approved by The College of Medicine, Veterinary and Life Sciences Ethics Committee (200160159).

Seven healthy control volunteers were recruited from University College London Hospital, Eastman Dental Hospital. The study was approved by East of England Cambridgeshire and Hertfordshire Research Ethics Committee (16/EE/0328).

All studies were conducted in full accordance with ethical principles, including the World Medical Association’s Declaration of Helsinki, as revised in 2000, and written informed consent was received from each participant.

**RNA sequencing**

A 4 mm punch biopsy tissue was obtained from ten participants with OFG alone (no concurrent CD) by a dedicated member of the clinical care team. The biopsy sample was placed in RNAlater^®^ (Life Technologies, UK) prior to RNA extraction using the RNeasy Fibrous Tissue Kit, as per the manufacturer’s instructions (Qiagen, UK). RNA quantity was assessed using a QUbit^TM^ 3.0 (ThermoFisher Scientific, UK). RNA quality was assessed using a Bioanalyser (Agilent, USA). All samples were confirmed to have a total RNA concentration >20ng/μl and a RIN score >7.0. Total RNA was sent to Novogene (Novogene, UK) for sequencing. RNA sequencing and bioinformatics analysis was performed using in-house Perl scripts, following the workflow shown in Figure 1. The data has been deposited in NCBI’s Gene Expression Omnibus and are accessible through GEO Series accession number GSE320069

(https://www.ncbi.nlm.nih.gov/geo/query/acc.cgi?acc=GSE320069).

**
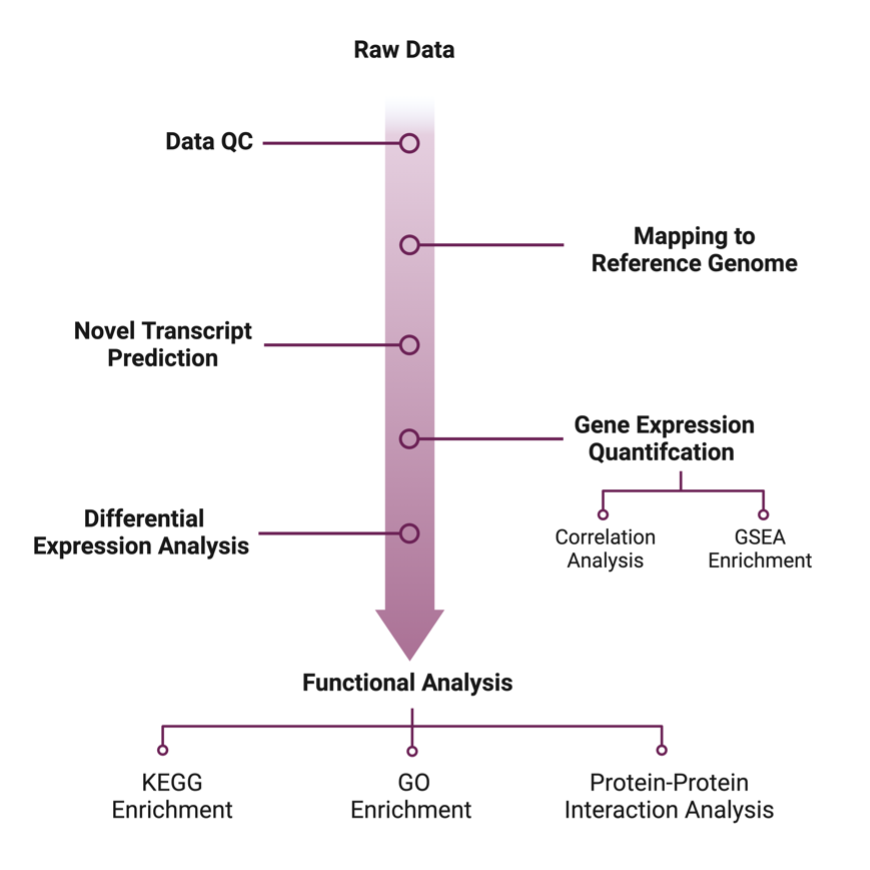
**

**Figure 1:** The diagram shows the bioinformatics pipeline for data analysis used by Novogene (Novogene, UK). Raw sequences first underwent quality control checkpoint analysis to obtain high quality reads. The reads were then mapped to the human reference genome, and the aligned transcripts were assembled and quantified. Differentially expressed genes were determined and functional analysis performed using pathway enrichment analysis with different databases.

**Whole Exome Sequencing**

Genomic DNA was extracted from whole blood from seventeen participants with OFG alone (no concurrent CD) using the DNeasy Blood and Tissue kit (Qiagen, UK), following the manufacturer’s instructions. Indexed paired-end libraries were prepared using the BGI 59 Mb Exome Enrichment Kit, and 2 × 150 base pair (bp) sequencing was performed on the Illumina HiSeq 2000 system (BGI, Hong Kong, China). Read alignment and variant calling of the exomes was performed simultaneously with approximately 5000 other exomes by an in-house NGS analysis pipeline. Reads were aligned to the human reference genome using hg38 by Novoalign (version 3.02.08) (2) and variants were called by the Genome Analysis Toolkit (GATK) according to best practice (3). Variants were annotated by the Variant Effect Predictor (version 76) (4). To identify potentially damaging variants the following filters were used: (i) MAF < 0.05 in the Non-Finnish European populations of the Exome Aggregation Consortium (ExAC)18 and Gnomad_NFE18, and MAF < 0.05 in Kaviar and (ii) Non-synonymous and not missense (e.g. stop gain, frameshift etc.), or missense and predicted damaging by at least one of SIFT, PolyPhen-216, Condel17, CAROL or CADD (≥ 20). Mutations that were identified in the same gene spanning the same region and mutations not identified in the exome database were removed from the analysis.

**Salivary biomarker analysis by Proximity extension assay**

Unstimulated saliva samples were collected from thirty participants with OFG and thirty healthy volunteers using the SalivaBio passive drool method (Salimetrics, UK). The saliva was freeze/thawed to break up mucin and centrifuged for 15 minutes at 3,000 rpm at 4 ^o^C to separate the cellular debris. The salivary immunoregulatory protein profile was determined using proximity extension assay (PEA) analysis by Olink Proteomics (Uppsala, Sweden) as previously described (5). Analysis was performed using two panels: the ProSeek Multiplex Inflammation panel (IFN1) (v.3012) and the ProSeek Immune Response panel (IR1) (v.3201). In combination this allowed for interrogation of salivary levels of 184 different proteins associated with the immune response (4 proteins were present on both panels; CCL11, IL-5, IL-6, IL-10).

After quality control checks, 1 OFG and 1 healthy control sample were removed from the analysis. For the remainder, proteins which were detected in <50% of samples were first interrogated separately for differences between groups. If no statistically significant differences between the groups were determined, then the proteins were excluded from further analysis.

**Salivary biomarker analysis by Enzyme-linked immunosorbent assay**

To validate the PEA data, ELISA analysis for salivary levels of IL-6, CCL3 and CXCL9 were performed using commercially available ELISA kits (Invitrogen, UK). The ELISA was performed in a 96 well plate format, according to the manufacturer’s instructions, and the absorbance of each well determined immediately at 450 nm on a FLUOstar Omega microplate reader (BMG Labtech, Buckinghamshire, UK).

Determination of salivary levels of calprotectin (MRP8/14; S100A8/9) was performed using a commercially available ELISA kit routinely used in clinical diagnostic labs in the West of Scotland (Buhlmann Laboratories AG, Switzerland). The ELISA was performed in a 96 well plate format, according to the manufacturer’s instructions, and the absorbance of each well determined immediately at 450 nm on a FLUOstar Omega microplate reader (BMG Labtech, Buckinghamshire, UK). This kit is designed for faecal calprotectin testing and so validation and quality control with human saliva samples was conducted in accordance with previously published methods (6).

**Microbiome analysis**

Unstimulated saliva samples from thirty-two participants with OFG and thirty-two healthy participants were collected using the SalivaBio passive drool method (Salimetrics, UK). Up to 2 ml of saliva was collected and the Salivabio tubes were frozen at -20 ^o^C immediately following collection. The sample was freeze/thawed to break up mucin and centrifuged for 15 minutes at 3,000 rpm at 4 ^o^C to pellet the microbial cells.

Microbial DNA was extracted from pelleted samples as previously described (7). Bacterial PCR amplicon libraries were created using primers targeting the V1-3 region of the bacterial 16S rRNA gene. Amplicons were sequenced using the Illumina MiSeq platform (250 nucleotides, paired-end) by Novogene (Novogene, UK). The paired-end reads were quality-filtered and processed into an OTU table with taxonomic annotation, as described previously described (7). Bacterial taxa were partitioned into either core and satellite taxa, based upon their prevalence and relative abundance across participant samples within each group (8). Categorisation of microbiota into core and satellite taxa was performed as it reveals important aspects of cross-sectional or longitudinal taxa-abundance distributions that would be neglected without such a distinction (8, 9). The sequencing data is available at PRJNA1429289 Temporary Submission ID: SUB16015641.

**Statistical analysis**

For clinical and demographic data differences were tested using a one-way ANOVA or the Pearson χ^2^ test.

For the RNA sequencing analysis, gene expression is presented as p adjusted values (padj) using the Benjamini and Hochberg's approach for controlling false discovery rate. Genes with a padj <=0.05 found by DESeq2 were considered statistically significant.

For the PEA data, significant differences in NPX values were adjusted for false discovery through the original false discovery rate method of Benjamini and Hochberg, with the false discovery rate set at 5%. Sample clustering was visually investigated using volcano plots and principal component analysis (PCA). Protein interactions between the groups were identified using the STRING database. SPSS (version 25.0; IBM Corporation, Armonk, NY).

For the microbiome data, regression analysis, coefficients of determination (*r*^2^), degrees of freedom (df), *F*-statistic and significance (*P*) were calculated using XLSTAT v2018.1 (Addinsoft, Paris, France). Fisher’s alpha index of diversity was calculated in PAST v4.13 (<http://folk.uio.no/ohammer/past>). This measure of diversity is relatively unaffected by variation in sample size, and completely independent if sequence reads per sample > 1000 (33). Significant differences in diversity between groups were determined using Kruskal-Wallis tests in conjunction with the post hoc Dunn test and performed in XLSTAT. Microbiota compositional similarity was measured using the Bray-Curtis index of similarity. Permutational multivariate analysis of variance (PERMANOVA) with Bonferroni correction was used to test for significance in microbiota composition and performed in PAST.

GraphPad Prism 7 (version 7.04; GraphPad Software Inc., La Jolla, CA) was used for other statistical analysis and the graphical presentation of results.

**Materials and Methods References**

1. White A, Nunes C, Escudier M, Lomer MC, Barnard K, Shirlaw P, et al. Improvement in orofacial granulomatosis on a cinnamon- and benzoate-free diet. Inflammatory bowel diseases. 2006;12(6):508-14.

2. Pontikos N, Yu J, Moghul I, Withington L, Blanco-Kelly F, Vulliamy T, et al. Phenopolis: an open platform for harmonization and analysis of genetic and phenotypic data. Bioinformatics. 2017;33(15):2421-3.

3. McKenna A, Hanna M, Banks E, Sivachenko A, Cibulskis K, Kernytsky A, et al. The Genome Analysis Toolkit: a MapReduce framework for analyzing next-generation DNA sequencing data. Genome Res. 2010;20(9):1297-303.

4. McLaren W, Gil L, Hunt SE, Riat HS, Ritchie GR, Thormann A, et al. The Ensembl Variant Effect Predictor. Genome Biol. 2016;17(1):122.

5. Sun BB, Chiou J, Traylor M, Benner C, Hsu YH, Richardson TG, et al. Plasma proteomic associations with genetics and health in the UK Biobank. Nature. 2023;622(7982):329-38.

6. Jaedicke KM, Taylor JJ, Preshaw PM. Validation and quality control of ELISAs for the use with human saliva samples. J Immunol Methods. 2012;377(1-2):62-5.

7. O'Donnell LE, Robertson D, Nile CJ, Cross LJ, Riggio M, Sherriff A, et al. The Oral Microbiome of Denture Wearers Is Influenced by Levels of Natural Dentition. PLoS One. 2015;10(9):e0137717.

8. Cuthbertson L, Walker AW, Oliver AE, Rogers GB, Rivett DW, Hampton TH, et al. Lung function and microbiota diversity in cystic fibrosis. Microbiome. 2020;8(1):45.

9. Cuthbertson L, Hatfield L, Gavillet H, Hardman M, Marsh R, Rivett DW, van der Gast C. Species turnover within cystic fibrosis lung microbiota is indicative of acute pulmonary exacerbation onset. Microbiome. 2025;13(1):140.
