# Supplementary material for: Investigations into the aetiopathogenesis of orofacial granulomatosis using multiple omics technologies reveal a potential role for B cells
Source: Clin Transl Med. 2026 May 12;16(5):e70689. doi: 10.1002/ctm2.70689 (PMC13162125; doi:10.1002/ctm2.70689)
Supplement: Supplementary file 2 — Supporting Information [file CTM2-16-e70689-s001.docx]

**Supplementary Table 1**

**Demographics:** Baseline characteristics according to final diagnostic category including age, gender, and smoking status. *Shisha

**Clinical scoring:** Comparison of mean rank site score and activity scoring for patients with orofacial granulomatosis solely (OFG alone) and orofacial granulomatosis with coexisting Crohn's disease (OFG + CD) based on a previously described standardized assessment of the oral cavity to characterise the number of sites affected, and the type of inflammation involved (see Supplementary Materials and Methods)

***Haematological parameters:*** *Select hematological markers of inflammation determined from blood tests required as part of their routine diagnostic work up.*
