# Supplementary material for: Investigations into the aetiopathogenesis of orofacial granulomatosis using multiple omics technologies reveal a potential role for B cells
Source: Clin Transl Med. 2026 May 12;16(5):e70689. doi: 10.1002/ctm2.70689 (PMC13162125; doi:10.1002/ctm2.70689)
Supplement: Supplementary file 4 — Supporting Information [file CTM2-16-e70689-s007.docx]

**SUPPLEMENTARY TABLE 3**

**T**he top 50 significantly downregulated genes in biopsy tissue of participants with orofacial granulomatosis compared to healthy control biopsy tissue as determined by RNAseq. The full data can be found in NCBI’s Gene Expression Omnibus and are accessible through GEO Series accession number GSE320069 (https://www.ncbi.nlm.nih.gov/geo/query/acc.cgi?acc=GSE320069).
