# Supplementary material for: Investigations into the aetiopathogenesis of orofacial granulomatosis using multiple omics technologies reveal a potential role for B cells
Source: Clin Transl Med. 2026 May 12;16(5):e70689. doi: 10.1002/ctm2.70689 (PMC13162125; doi:10.1002/ctm2.70689)
Supplement: Supplementary file 5 — Supporting Information [file CTM2-16-e70689-s004.docx]

**SUPPLEMENTARY TABLE 4**

Whole-exome sequencing was performed on participants with orofacial granulomatosis (OFG) without co-existing Crohn’s disease (n = 17) (see Supplementary Materials and Methods). The table summarises the most frequent protein-altering variants identified, including missense, frameshift, splice-region, and stop-gained mutations, ranked by allele frequency (AF). Variant frequencies were compared against the ExAC reference database (~60,000 human exome sequences) to assess rarity and potential enrichment in the OFG cohort. Predicted functional impact of each variant is indicated by the Combined Annotation-Dependent Depletion (CADD) score (70), indicating the likelihood of a variant being pathogenic (<10 likely benign, 10-30 possibly deleterious, >30 highly deleterious).

**
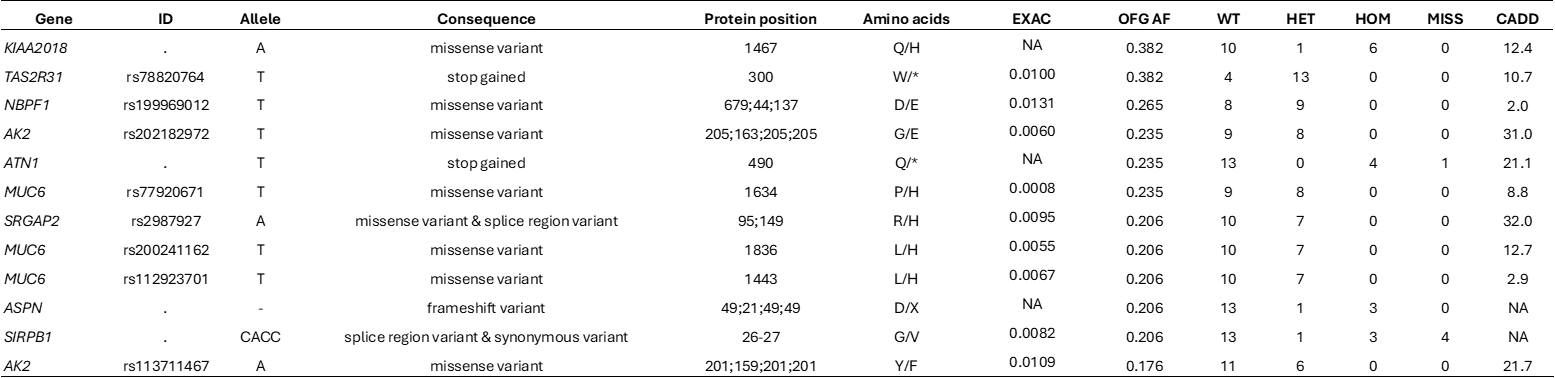
**

The most frequent mutations associated with OFG are found in the *KIAA2018* gene (allelic frequency = 0.382 in OFG, no data available for a non-finish European population) and *TAS2R31* gene (allelic frequency = 0.382 in OFG compared to 0.0100 in a non-finish European population). However, based on Combined Annotation-Dependent Depletion (CADD) score, a predicted measure of variant deleteriousness, mutations in the *SRGAP2* (CADD = 32) and *AK2* (CADD = 31) genes may have a greater potential impact on disease. Furthermore, WES analysis identified several mutations in the *MUC6* gene to be tentatively associated with OFG.
