# Supplementary material for: Investigations into the aetiopathogenesis of orofacial granulomatosis using multiple omics technologies reveal a potential role for B cells
Source: Clin Transl Med. 2026 May 12;16(5):e70689. doi: 10.1002/ctm2.70689 (PMC13162125; doi:10.1002/ctm2.70689)
Supplement: Supplementary file 6 — Supporting Information [file CTM2-16-e70689-s005.docx]

**SUPPLEMENTARY TABLE 5**

Significantly upregulated immunoregulatory proteins in the saliva of participants with orofacial granulomatosis compared to healthy control participants as determined by using proximity extension assay (PEA) analysis by Olink^®^ Proteomics (Uppsala, Sweden)
