# Supplementary material for: Investigations into the aetiopathogenesis of orofacial granulomatosis using multiple omics technologies reveal a potential role for B cells
Source: Clin Transl Med. 2026 May 12;16(5):e70689. doi: 10.1002/ctm2.70689 (PMC13162125; doi:10.1002/ctm2.70689)
Supplement: Supplementary file 7 — Supporting Information [file CTM2-16-e70689-s003.docx]

**SUPPLEMENTARY TABLE 6**

Similarity of percentages (SIMPER) analysis between microbiota within orofacial granulomatosis (OFG) and healthy participants.

|  |  |  | **Mean % abundance** | |
| --- | --- | --- | --- | --- |
| **Taxon** | **Contrib. %** | **Cumulative %** | **Healthy** | **OFG** |
| *Streptococcus*_1 | 13.35 | 13.35 | 26.70 | 21.70 |
| *Neisseria*_1 | 7.00 | 20.34 | 3.18 | 6.54 |
| *Streptococcus*_2 | 6.97 | 27.31 | 8.44 | 7.08 |
| *Haemophilus parainfluenzae* | 5.56 | 32.87 | 3.75 | 5.24 |
| *Rothia* | 5.34 | 38.21 | 8.40 | 6.54 |
| *Streptococcus*_3 | 4.35 | 42.56 | 4.92 | 2.98 |
| *Actinomyces odontolyticus* | 4.00 | 46.56 | 5.19 | 2.52 |
| *Prevotella melaninogenica* | 3.97 | 50.53 | 2.37 | 3.01 |
| *Rothia mucilaginosa* | 3.35 | 53.88 | 3.78 | 2.74 |
| *Veillonella*_1 | 3.29 | 57.17 | 3.49 | 4.42 |
| *Prevotella histicola* | 2.59 | 59.76 | 0.71 | 2.41 |
| *Lactobacillales* | 2.52 | 62.28 | 3.13 | 3.49 |
| *Gemella* | 2.31 | 64.59 | 2.54 | 2.89 |
| *Veillonella*_2 | 2.02 | 66.61 | 1.69 | 2.16 |
| *Actinomyces* | 1.96 | 68.57 | 2.32 | 1.35 |
| *Porphyromonas* | 1.59 | 70.16 | 0.55 | 1.21 |
| *Acinetobacter guillouiae* | 1.38 | 71.54 | 0.0003 | 1.12 |
| *Neisseria*_2 | 1.34 | 72.88 | 0.30 | 1.17 |
| *Leptotrichia* | 1.31 | 74.20 | 0.42 | 1.16 |
| *Actinomyces graevenitzii* | 1.29 | 75.48 | 1.30 | 1.14 |
| *Fusobacterium periodonticum* | 1.10 | 76.58 | 0.62 | 1.07 |
| *Oribacterium* | 1.04 | 77.63 | 1.08 | 0.63 |
| *Atopobium* | 0.92 | 78.55 | 1.15 | 0.65 |
| *Veillonella*_3 | 0.89 | 79.44 | 0.90 | 0.98 |
| *Rothia dentocariosa* | 0.74 | 80.18 | 0.80 | 0.79 |

All taxa listed had core taxa status within each group, except *Acinetobacter guillouiae* which had satellite status in each instance. Percentage contribution (Contrib. %) is the mean contribution divided by mean dissimilarity across samples (39.9%). The list of species is not exhaustive, so cumulative percent does not sum to 100%. Given is within category mean percent abundance for taxa. ASV numbers have been used to differentiate between taxa within the same genus. Given the length of the ribosomal sequences analysed species identities should be considered putative.
